# The Research Advance of Cell Bridges *in vitro*

**DOI:** 10.3389/fbioe.2020.609317

**Published:** 2020-11-24

**Authors:** Qing Zhang

**Affiliations:** ^1^College of Sericulture, Textile and Biomass Sciences, Southwest University, Chongqing, China; ^2^State Key Laboratory of Silkworm Genome Biology, Southwest University, Chongqing, China; ^3^Hunan Provincial Key Laboratory of Controllable Preparation and Functional Application of Fine Polymers, School of Chemistry and Chemical Engineering, Hunan University of Science and Technology, Xiangtan, China

**Keywords:** regeneration medicine, chemical pattern, cell bridges, topography, tissue engineering

## Abstract

The microenvironment in which cells reside *in vivo* dictates their biological and mechanical functioning is associated with morphogenetic and regenerative processes and may find implications in regenerative medicine and tissue engineering. The development of nano- and micro-fabricated technologies, three-dimensional (3D) printing technique, and biomimetic medical materials have enabled researchers to prepare novel advanced substrates mimicking the *in vivo* microenvironment. Most of the novel morphologies and behaviors of cells, including contact guidance and cell bridges which are observed *in vivo* but are not perceived in the traditional two-dimensional (2D) culture system, emerged on those novel substrates. Using cell bridges, cell can span over the surface of substrates to maintain mechanical stability and integrity of tissue, as observed in physiological processes, such as wound healing, regeneration and development. Compared to contact guidance, which has received increased attention and is investigated extensively, studies on cell bridges remain scarce. Therefore, in this mini-review, we have comprehensively summarized and classified different kinds of cell bridges formed on various substrates and highlighted possible biophysical mechanisms underlying cell bridge formation for their possible implication in the fields of tissue engineering and regenerative medicine.

## Introduction

Cell–cell contact (Ilkka et al., 2018), bioactive factors (Jiali et al., 2019) and extra-cellular matrix (ECM) (Ramirez-San et al., 2017) provide biomechanical and biochemical cues that are crucial for cell phenotype and function. The interaction of extracellular matrix (ECM) with cells is predominantly mediated through focal adhesion (Ray et al., 2017; Yu et al., 2020). However, cells suspend easily in the absence of attachment to ECM due to the discontinuity of adhesion sites and the complexity and heterogeneity of ECM geometrical cues, which varies with the cell type and the Rac activation (Guillou et al., 2008). Particularly, cells have the capacity to sense and transduce the chemical and physical properties of ECM to their cytoskeletal structures to bridge across the non-adhesion areas and maintain the mechanical stability and integrity of tissue (Thery, 2010), as observed in wound healing (Vedula et al., 2015), regeneration (Goldshmit et al., 2012) and development (Whong, 1931).

Decades of research have indicates that the microenvironment of cells plays a crucial role in regulating its behaviors and function both *in vivo* and *in vitro* (Carrel and Burrows, 1911). Since the 1980s, the introduction of photolithographical techniques, initially developed for the microelectronic industry, into the cell culturing field has led the development of patterned substrates for cell analysis and culture (Curtis and Varde, 1964). The majority of cell types can adapt their adhesion, morphology, migration, cytoskeleton and/or genome according to the chemical and topographic patterns on artificial substrates mimicking the native ECM microenvironment (Ahmed and Ffrench-Constant, 2016; Ma et al., 2018). Notably, some novel cell morphologies which in part dictates the cell such as contact guidance (Nguyen et al., 2016; Ray et al., 2017) and cell bridges (Goldner et al., 2006; Zhang et al., 2015b), have been observed on the patterned substrates at micro and nano scale. While contact guidance has been extensively investigated in regeneration medicine and tissue engineering (Kamyar et al., 2018; Tonazzini et al., 2019), studies on cell bridges observed on chemical pattern substrates and some nano/micro topographical substrates remain scarce.

Although the traditional two-dimension (2D) culture system is extensively used as a valuable tool for cell-based studies, it exhibits several limitations (Liu et al., 2018), such as abnormal structural characteristics, mechanical constraints, not being able to accurately mimic the natural microenvironment where cells reside in tissues and interactions between cells. Thus, as an alternative to the technical limitations of the 2D culture system, a 3D culture system with the advantage of mimicking the microenvironment of natural ECM is preferred for regenerative medicine and tissue engineering research (Joyce et al., 2018; Lin et al., 2019). Besides, 3D scaffolds can provide a better representation of the natural tissue architecture, chemical composition, and mimic cell communication between the cell and microenvironment (Hassani et al., 2020; Hou et al., 2020). More recently, scaffold-based 3D assembly of cells has been applied and used as a supporting structure to guide cell adhesion, growth, differentiation, and arrangement in a manner analogous to developmental processes or tissue repair (Liu et al., 2018). Thus, 3D culture systems overcoming the obstacles arising from traditional 2D culture systems have emerged as pioneering methodology and extensively utilized for stem cell research, regenerative medicine studies, and tissue engineering (Yuan et al., 2018). On different types of 3D substrates, such as microspheres, porous scaffolds or 3D printing scaffolds (Jamal et al., 2010), various cell types span across different surfaces of scaffold and form cell bridges with single-cell or collective cells (Alvim Valente et al., 2018).

With the recent advancement of tissue engineering, cell biology, and biomaterials science, various substrates or scaffolds with more defined topographic (Zhao et al., 2018) and chemical (Na et al., 2017) pattern are prepared to mimic the natural cell niche in tissue in order to guide the cells behavior. Contact guidance (Zhang et al., 2015a; Tonazzini et al., 2019), cell adhesion (Vasiliki and Triantafyllos, 2018), differentiation (Yao et al., 2016; Zhang et al., 2019) and gene expression (Qiongfang et al., 2018) have been paid much attention and investigated extensively; however, cell bridges, a phenomenon as consistent as contact guidance remain to be completely elucidated. As an essential behavior of cells, cell bridges may play crucial role in organization of cells, migration and development. Therefore, particular attention should be paid to these cell bridges in cell biology, regenerative medicine and embryonic development. In this mini-review, we comprehensively summarized and classified different kinds of cell bridges formed on various substrates; besides, the process of formation, possible mechanism and cues of cell bridges will be outlined and highlighted. The effects of the formation of cell bridges on cell proliferation and/or differentiation will be reviewed. We will also discuss the need for investigations of cell bridges. This article will provide critical overview of cell bridges for their possible implications in the fields of tissue engineering, biology and regenerative medicine and will be helpful in expanding our understanding of cell behaviors.

## Approaches of Cell Bridging

Cells can span across non-adhesion areas or concave and suspend their body in air irrespective of chemical pattern or topography both *in vivo* and *in vitro*. In this section, the approaches of cell bridging *in vitro* will be summarized, discussed, and classified.

**FIGURE 1:**
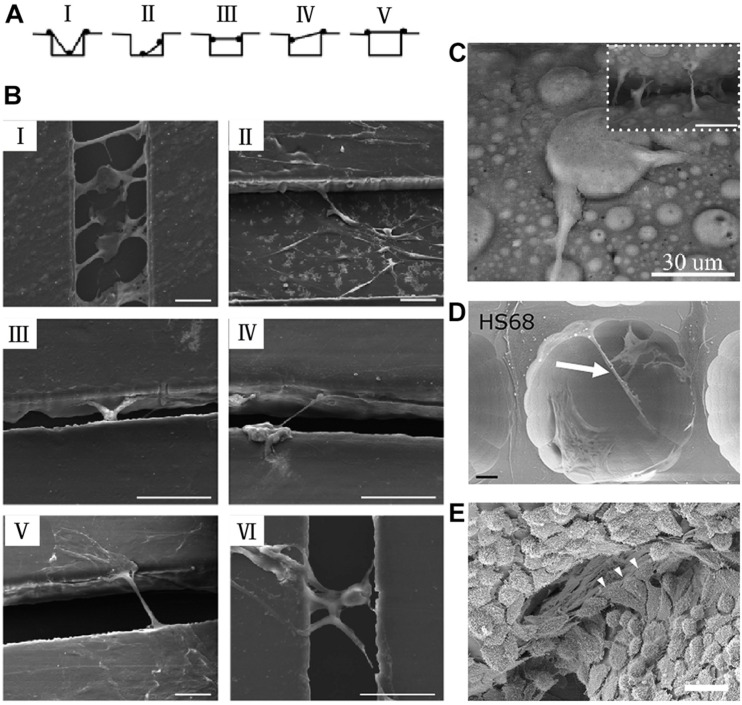
Various morphologies of cell bridges resulting from topographic cues. The sketch maps **(A)** and SEM photos **(B)** of various types of human mesenchymal stem cells (hMSCs) bridges observed on micro-grooved substrates. In panels **(AI,BI)**, cells contacted with substrates at bottom and two side walls. In panels **(AII,BII)**, cells adhered bottom and one side wall. In panels **(AIII–V,BIII–V)**, cells suspended above grooves by climbing two side walls, one side wall and the other side plateau or two side plateau, respectively. Collective cells bridges crossing grooves as shown in panel **(BVI)**. From Zhang et al. (2015b). Copyright 2015 RSC. **(C)** mouse mesenchymal stem cells (mMSCs) bridges spanning over two microspheres. From Cheng et al. (2014). Copyright 2014 RSC. **(D)** Fibroblasts bridges stretching inside the etched features. From Nikkhah et al. (2010). Copyright 2010 Elsevier. **(E)** Fibroblasts bridges across the gap between two neighboring rigid panels. From Jamal et al. (2010). Copyright 2010 Elsevier. Scale bars represent 50 μm in panel **(B)**, 20 μm in panel **(D)**, and 30 μm in panel **(E)**.

Topography, as a critical factor of microenvironment, impose specific mechanical boundary conditions to cells, has engrossed increasing attentions over a few decades due to its influence on cell mechanics, function, polarity, and architecture (Abagnale et al., 2017). Various topographies with different morphology and size (from nanometer, sub-micrometer to micrometers) have been prepared to investigate the phenomenon and mechanism of cell behavior and function (Nguyen et al., 2016; Ribeiro et al., 2019). Several kinds of cells spreading and morphology have been reported on diverse topographic substrates and can be roughly classified into three patterns: (1) growing along the topography, as represented by Xenopus tissue on micropost arrays (Song et al., 2015) and neurons on ring-shaped nanopillar arrays (Xie et al., 2010); (2) engulfing the topography, as shown by neurons on gold-spine electrode (Hai et al., 2010); (3) spanning across topography, as depicted by primary hippocampal neurons on pillar arrays (Park et al., 2016a,b). Furthermore, cell bridges containing a single cell or multiple cells on groove substrates have been summarized and described by five strategies (Figures 1AI–V,B–V) depending on the interaction between cell/cell and topographic substrates, as illustrated in Figure 1A (sketch maps) and Figure 1B (scanning electron microscope photos) (Zhang et al., 2015b). In the first strategy as shown in Figures 1AI,BI, cells located at the bottom of grooves with other parts of their body adhering the two side walls. In the second strategy as shown in Figures 1AII,BII, cells suspended in air with their body adhering the bottom of grooves and one side wall. In other strategies, cells bridged across grooves with their body grasping the two side walls (Figures 1AIII,BIII), one side wall and the other side plateau (Figures 1AIV,BIV) or two side plateau (Figures 1AV,BV). Sometimes, cells combined together to bridge grooves as shown in Figure 1BVI. In this section, cell bridges constituting different kinds of cells, i.e., mesenchymal stem cells (MSCs), neurons, on a variety of topographic surfaces with different structure and biochemical compositions, will be classified according to the strategies shown in Figures 1A,B, and will be summarized as presented in Table 1. Typical cell bridges between two neighboring microspheres, two surfaces of a scaffold, and a cavity structure are shown in Figures 1C–E, individually (Jamal et al., 2010; Nikkhah et al., 2010; Cheng et al., 2014).

**TABLE 1:** Cell bridges formation on various topographic substrates.

**Cells**	**Single-cell (S) or multi-cells (M)**	**Approaches of cell bridging**	**Substrate composition**	**Morphology of topography**	**References**
NIH-3T3 (a mouse embryonic fibroblast line)	S	V	Silicon	Triangular pores with 3∼20 μm long sides	Sunami et al., 2014
3T3	S	II,V	Polydimethylsiloxane (PDMS)	Micropillars	Ghibaudo et al., 2009
hMSC	S, M	II,V	Alumina ceramic	Micropillars	Lauria et al., 2016
Mouse embryonic fibroblasts (MEFs)	S	V	PDMS	Pillars with 0.5 and 2 μm distance	Ghassemi et al., 2012
Cardiomyocytes	S	V	Coated with poly-L-Lysine	Three-dimensional microstructure scaffolds	Klein et al., 2010
Human endothelial cell	S	V	PDMS	Microgroove with 2 and 10 μm width	Sales et al., 2019
Epithelial cell	M	II,V	PDMS	Large-scale curvature	Broaders et al., 2015
Epithelial cell	S	V		70 nm × 400 nm × 600 nm (width × pitch × depth) and 1900 nm × 4000 nm × 600 nm grating	Teixeira et al., 2006
Neuron	S	II,V		Pillar with 6 μm distance	Park et al., 2016
C2C12 (a mouse myoblast cell line)	S, M	I, II, V	PDMS, PLLA (Poly L-lactic acid), PEOT/PBT (poly(ethylene oxide)/poly(butyleneterephtalate))	Pillar with 4.5 and 10 μm height and 2 and 5 μm space	Papenburg et al., 2010
Human fibroblast cell (HS68) and cancer cell	S	II, V	Silicon	Microchamber	Nikkhah et al., 2010
MSCs	S	V	Polyimide	650 nm grooves	Abagnale et al., 2015
MSCs	S	II, V	Chitosan (CS)	Micro-hills: 10.1–13.0 μm diameter with 4.2 ± 3.29 μm space and 4.86–22.9 μm diameter with 13.9 ± 10.87 μm space	Yang et al., 2011
hiPSK3 cell (Human iPSK3 cells, derived from human foreskin fibroblasts transfected with plasmid DNA encoding reprogramming factors OCT4, NANOG, SOX2 and LIN28)	S	V	PDMS	560 nm height grating with 500 nm space	Song et al., 2016
oligodendrocyte-type 2 astrocyte (O-2A) progenitors	S	V	quartz	4 μm grooves	Webb et al., 1995
PC12 (an adult rat adrenal medulla pheochromocytoma Cell lines)	S	V	Conductive polypyrrole (wPPy)		Aufan et al., 2015
Hippocampal murine neural progenitor cell	S	V	PDMS	2 μm × 2 μm × 2 μm and 2 μm × 2 μm × 4 μm grooves	Jie et al., 2014
mMSC	S	II	PLGA/PCL (poly(lactic-co-glycolic acid)/polycaprolactone)	Between two microspheres	Cheng et al., 2014
Fibroblast	S	II	Au-photoresist	Between the two adjacent rigid panels in a 3D scaffold	Jamal et al., 2010
Epithelial Madin-Darby canine kidney cells (MDCK-)	M	II	Silicon nitride	0.8 μm pores scaffolds	Rother et al., 2015
Neuron	S	II		Nano-line with 75 nm height and 3 μm width	Baranes et al., 2019
NIH-3T3	S	V	PUA(polyurethane Acrylate)	Nanopillars	Kim et al., 2017
Gingival fibroblast-like cells	M	V	PCL, PCL70/PLGA30, PLGA	15 and 20 μm grooves	Alvim Valente et al., 2018

**FIGURE 2:**
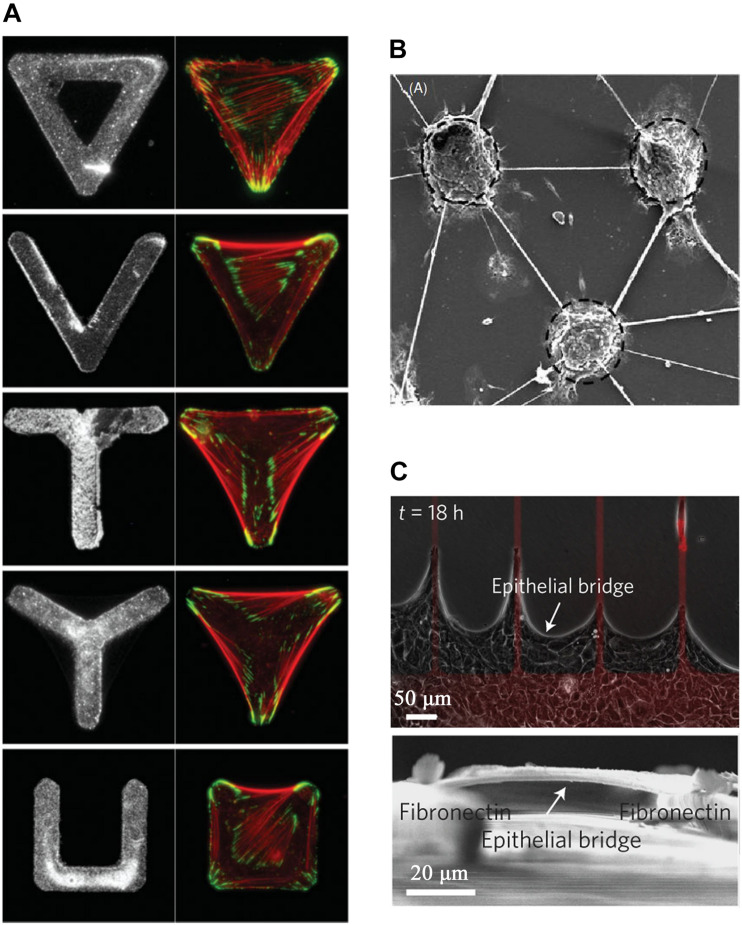
Various morphologies of cell bridges spanning non-adhesive areas on chemical micropatterns. **(A)** Single cell bridges were directed by various adhesive shapes (triangle, “V,” “T,” “Y,” and “⊔”) coated by homogeneous fibronectin for cell adhesion. The edge length of the triangle is 46 μm. From Théry et al. (2010). Copyright 2006 Wiley-Liss. **(B)** Axons and dendrites bridges connecting 100 μm adhesive islands on which neurons clusters adhered. From Sorkin et al. (2006). Copyright 2006 Institute of Physics Publishing. **(C)** Epithelial collective cells bridges suspending over non-adhesive areas with a part of cells adhesion on fibronectin strips. From Vedula et al. (2014b). Copyright 2013 Nature Publish Group.

**FIGURE 3:**
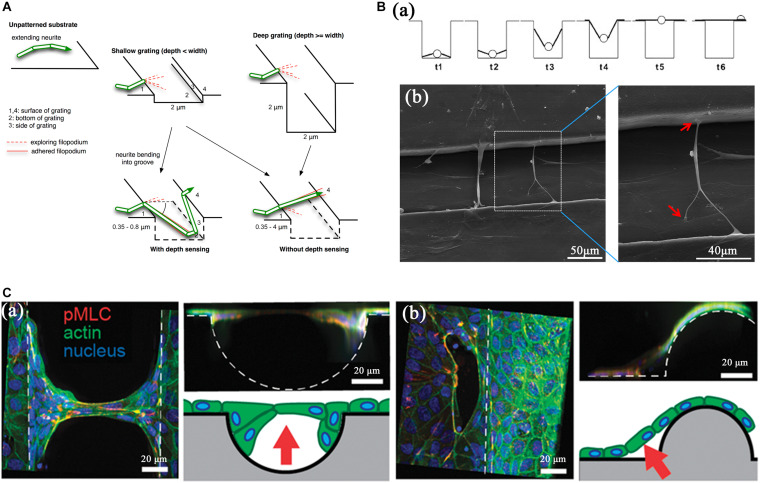
Various processes for the formation of cell bridges caused by topographic cues. **(A)** A schematic diagram of neurite bridges formation process with depth sensing, during which cells stretched themselves across the groove when depth ≥ width. From Jie et al. (2014). Copyright 2014 Elsevier. **(B)** Cells initially at the bottom, extended to the groove walls and bridged across two adjacent plateaus. Panel **B(a)** is a diagrammatic drawing of this process and panel **B(b)** is SEM photos about this strategy. Panel **B(a)** from Goldner et al. (2006). Copyright 2006 Elsevier. Panel **B(b)** from Zhang et al. (2015b). Copyright 2015 RSC. **(C)** Tissues raised from negative curvature regions by the contractility force of neighboring cells. Lifted tissue at channels and bridges were shown in panel **C(a,b)** individually. From Broaders et al. (2015). Copyright 2015 Oxford University Press.

The adhesion sites, mediating the attachment of cells to ECM, are not continuous in the microenvironment of cells, and its spatial distribution is predominantly dependent on the biochemical composition pattern of the microenvironment (chemical microenvironment), which is dictated by the location and orientation of ECM fibers and the positions of adjacent cells (Thery, 2010). Consistent with the topographic microenvironment, chemical cues also play a crucial role in the regulation of cellular differentiation, gene expression, and functions (Xing et al., 2019). Chemical micro-patterns are prepared to mimic the chemical microenvironment *in vivo* to accurately investigate the cell behavior and mechanism based on cell adhesions (Tu et al., 2017). On these artificial chemical micro-patterns, in addition to being confined to adhesion micro-areas, cells can also span across the non-adhesion area and grow into cell bridges identical to axon (Sorkin et al., 2006) and epithelium bridges (Vedula et al., 2014b) emerged during wound healing *in vivo*. Three key patterns have been identified for the formation of cell bridgs on chemical micropattern (Figure 2): (1) single-cell spreading on specific chemical micropattern with parts of cell body suspended on non-adhesion areas (Théry et al., 2010; Figure 2A); (2) multiple-cells spanning over a non-adhesion area while communicating each other (Vedula et al., 2014b; Figure 2C); (3) axon and dendrite spanning over non-adhesion areas without suspending cell bodies (Sorkin et al., 2006; Figure 2B). Cell bridges arising on chemical micropattern have been summarized in Table 2.

**TABLE 2:** Cell bridges on various chemical micropattern substrates.

**Cells**	**Single-cell (S), multi-cells (M) or axon and dendrite (A&D)**	**Features of chemical micropattern**	**References**
HeLa cell (a human cervical carcinoma cell line)	S and M	A honeycomb network of adhesion	Albert and Schwarz, 2016c
HaCaT cell (a spontaneously transformed aneuploid immortal keratinocyte cell line from adult human skin) and MDCK cell	M	100 and 200 μm diameter non-adhesion gaps	Vedula et al., 2015
HaCaT cell and MDCK cell	M	10-μm-wide fibronectin strips separated by either 120 or 400 μm	Vedula et al., 2014a
Neuron	A&D	PDL and CNT island with 150–400 μm separation	Sorkin et al., 2006
HEK293 cells (a human embryonic kidney cell line)	S	Parallel stripes, T-shape and hexagon	Suffoletto et al., 2015
Epithelial cell	M	Converging, parallel, and diverging adherent paths	Hu et al., 2017
hTERT-RPE1 (a human retinal pigment epithelial cell line)	S	Frame, “V,” “T” and tripod micropatterns	Théry et al., 2010
Keratinocytes	M	Non-adhesive patch	Vedula et al., 2015
MDCK cell	M	Non-adhesive patch	Nier et al., 2015

## The Key Cues for Initiation of Cell Bridges Formation and the Extremities of Cell Bridges

Cellular morphology and organization are crucial for migration and differentiation of cells and tissue microarchitecture. Therefore, it becomes imperative to identify cues that initiate the formation of some typical morphology and organization of cells within the tissue for implication in tissue regeneration, morphogenesis, and engineering. In this section, some key cues for initiation of cell bridges formation and the extremities of cell bridges will be summarized and discussed. All content will be presented in two parts according to cell bridges directed by topography or chemical patterns.

According to the papers published until now, three primary cues have been identified to affect the formation of cell bridges on topographic substrates: (1) the characteristics of cells, including size, source, age of donor; (2) the features of topographic substrates, including size, porous structure, roughness, and pattern; (3) the wettability of substrates. Noticeably, cells naturally suspend parts of their body and adhere to substrates with the other parts of their body when they are cultured on topographic substrates with spacing between pillars or ridges of smaller (such as nano or sub-micro meter) than cell size, because cells prevent bending themselves to adapt to the uneven of topography (Ohara and Buck, 1979). Of note, the mechanical stress exerted on cells increases with increasing pattern size (Sunami et al., 2014). In contrast, the persistence length of cell filopodia bridging perpendicular to the grooves decreases with the increasing age of donors (Sales et al., 2019). If the space between pillars or ridges is comparable to the size of cells, then cells may span between neighboring pillars or ridges or be confined to the bottom of pillars, or a single ridge or groove. Nevertheless, on the deeper and narrower groove, it is easier for cells to bridge neighboring ridges (Braber et al., 1996, 2010; Curtis and Wilkinson, 1997). Furthermore, the ratio of groove width to depth remains crucial for forming cell bridges, and hMSCs only bridge across neighboring ridges when encountering grooves with a ratio of width to depth of less than two within the range of testing (Zhang et al., 2015b). The largest distance for cells to bridge can vary with the cell type and substrate size [ranging from 50 μm (Stevenson and Donald, 2009) to 200 μm (Zhang et al., 2015b)]. Cell bridges spanning across large distance areas are usually composed of multi cells or cell collective. Furthermore, the formation of multicellular epithelial bridges across negative curvature grooves with dozens of micrometers width has been found to be associated with the ratio of the cosine and sine of cell–cell and cell-substrate contact angle and cell type (Broaders et al., 2015). Noticeably, cancer cells prevent bridging and tend to adapt to the curved surfaces of the chamber due to their easy distortion caused by impaired cytoskeleton compared to fibroblast cells (Nikkhah et al., 2010). Moreover, the wettability of the material surface may trigger the transformation from spreading on top of the pillars to spreading on the bottom. Cell bridges forms over pillars with 4.5 μm height and 5 μm spacing on the PDMS surface; however, spread on the bottom of the surface with the same pillars on poly(ethylene oxide)/poly(butyleneterephtalate; PEOT/PBT) and poly L-lactic acid (PLLA) surfaces (Papenburg et al., 2010).

Similar to the formation of cell bridges on topography substrates, there are major three factors that affect the formation of cell bridges on chemical micropattern: (1) the characteristics of chemical micropattern, including size, shape, and distance; (2) the characteristics of cells, such as cell type, the contact force between each other; (3) the initial sites of cell localization. In general, a single cell is able to bridge across the non-adhesion areas on various chemical micropatterns, which usually enable the cell to adhere to specific areas on substrates. In this condition, cells usually suspend parts of their body and adhere to substrates with the other parts of their body when they are cultured on “T”- or “V”-shaped adhesion micropatterns; however, they adhere to round or square adhesion micropatterns with their whole body and turn to be round or square shape accordingly. In addition, cells can form a multicellular bridge to span across a non-adhesion area with a considerable distance. The adhesion force between cells, the distance between adhesion strips, and the width of adhesion strips remains crucial for the building of these multicellular bridges (Vedula et al., 2014b). Moreover, the increasing adhesion strip width and the decreasing spacing between adhesion strips are both beneficial to the formation of multicellular bridges. The critical distance between adhesion strips at which multicellular bridges form is limited to 200 μm (Vedula et al., 2014b). According to a study of artificial neural networks reported by Hanein et al., cell density influences the formation of the bridges composed of dendrites and axons (Sorkin et al., 2006). In this system, the maximal spanning distance of a bridge was reported to be as long as 400 μm (Sorkin et al., 2006). In contrast, Lehnert et al. (2003) reported that cells could span 3∼4 adhesive sites on adhesive square islands substrates if they are initially located non-adherent areas between adhesive squares, but not bridge across non-adhesive gaps if they initially centered on adhesive squares (Dirk, 2004).

## The Progress and Mechanism of Cell Bridges Formation

The characteristics of cells, such as cell membrane elasticity (Ohara and Buck, 1979), tensile forces from pulling cells (Vedula et al., 2014b), substantially affect the formation of cell bridges. Possible processes involved in the formation of cell bridges have been speculated by various investigators based on their results. These processes will be discussed separately, depending on the substrates (topography or chemical micropattern) on which cell bridges formed.

As the radical bending of the cell body is prevented by cell membrane elasticity or rigidity, so cells prefer bridging over the top of nanofeatures, neighboring ridges, or neighboring faces of a scaffold to adapt to the morphology of topographic substrates (Jie et al., 2014). Three different processes have been described to reveal the process of cell bridges formations (Figure 3). Jie et al. (2014) prepared a string of gratings to investigate the effect of the depth of micro-grating on the spreading of murine neural progenitor cells. Furthermore, to identify the relationship of neuronal elongation and alignment with grating depth, a quantitative model was established to explain the depth-sensing mechanism of the cells and predict bridging over the neighboring gratings (Figure 3A). Based on their hypothesis, when cells encounter a grating edge they first initiate filopodia to randomly probe the potential adhesion sites, then cells extend along with the gratings, adhere to the bottom of the groove, or bridge across the gap between neighboring gratings depending on the features of topography. Their results suggested that if the cytoskeleton contractility caused by the adhesion of filopodia to a substrate is strong enough to balance the microtubule bending resistance, cells will adhere to the bottom of the groove; if not strong enough, cells will favor extending along with gratings or bridging over grooves when they form long and sufficient number of horizontal filopodia. Different from this hypothesis, other researchers suggested the formation of cell bridges was driven by the higher site of cell which climbed along the groove walls to the adjacent plateaus (Goldner et al., 2006; Zhang et al., 2015b). Cells initially located at the bottom suspended themselves by the contractile force during their climbing along the wall to a higher site, as represented in Figure 3B(a). Previously, Gartner et al. reported that in epithelial Madin-Darby canine kidney (MDCK) cells, the majority of detachments occurred in the areas most significant concavity (center of channels and the convex ridges) (Broaders et al., 2015). According to their hypothesis (Figure 3C), the lateral contractile force generated by the surrounding tissue exhibited the potential to resist the adhesive forces centered at areas of greatest concavity areas raised the cells up.

**FIGURE 4:**
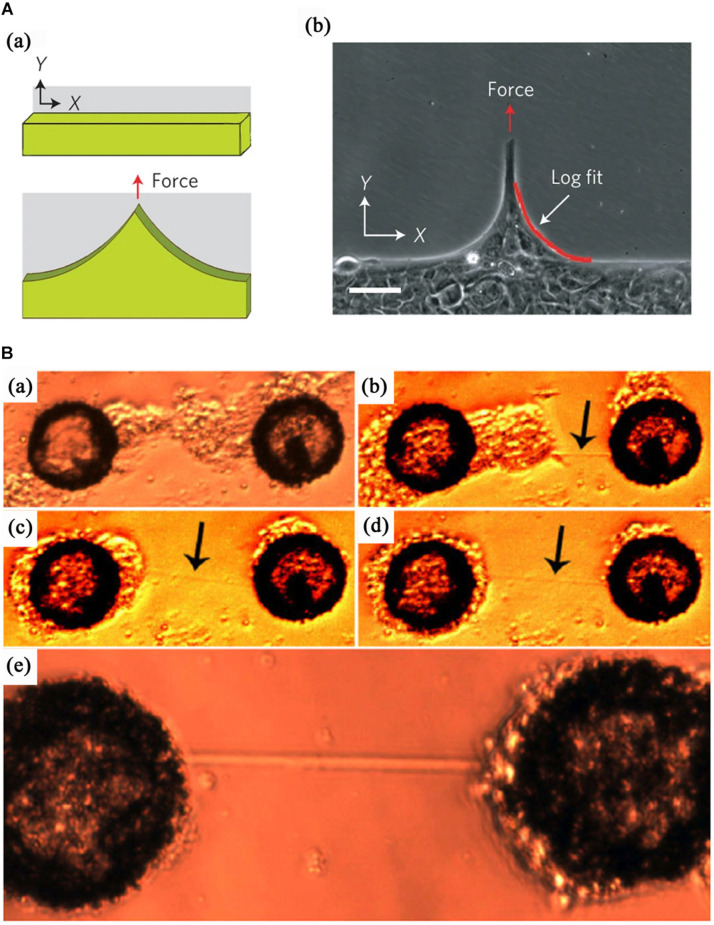
Two processes for the formation of cell bridges on chemical micropatterns. **(A)** The formation of multicellular bridges driven by the traction of cell migration along adhesive strips separated by non-adhesive regions. From Vedula et al. (2014b). Copyright 2013 Nature Publish Group. **(B)** Bridges composed of axons and dendrites resulting from the self-assembly of cells into clusters on separated adhesive islands. From Sorkin et al. (2006). Copyright 2006 Institute of Physics Publishing.

Consistent with topographic substrates, the formation of cell bridges on chemical micropatterns was resulted from the migration and interaction of cells. Sorkin et al. (2006) demonstrated a patterned neural network consisting of cell clusters and non-adherent bundles between them. Cells after being seeded on adhesion micropattern substrates formed cell clusters; subsequently, the cell clusters migrated and anchored at specific positions, which triggered the formation of dendrites and axons bridges spanning non-adherent areas and an artificial neural network, as shown in Figure 4B. Furthermore, epithelial bridges were easily formed when cell ensemble migrated on heterogeneous substrates (Albert and Schwarz, 2016a). More recently, Ladoux et al. reported multicellular bridges to span across non-adherent areas when human keratinocytes were allowed to migrate along surfaces micropatterned with alternating strips of non-adherent polymer and fibronection, as shown in Figure 4A (Vedula et al., 2014b). They hypothesized that the tensile forces transferred from the traction of cells on the fibronectin strips drove the formation of epithelial bridges. For a single cell bridging on “V” or “T” shapes, the geometry of adhesive substrates forces cells to reorganize their internal cytoskeleton and bridge non-adhesion site, which is characterized by reinforcement of peripheral actin bundles and formation of RhoA-dependent stress fibers (James et al., 2010; Théry et al., 2010; Rossier et al., 2014).

As mentioned earlier, some ambiguous hypotheses were raised to explain the process and mechanism of cell bridges formation, few of which consider and explain the underlying molecular and mechanotransduction mechanisms. In contrast, common theories, including polarization of F-actin due to mechanical restriction (Dunn and Heath, 1976), maximization of focal adhesion areas (Ohara and Buck, 1979), and actin polymerization resulting from discontinuities of substrates (Curtis and Clark, 1990), have also been proposed justify contact guidance. Recently, Deshpande and Bouten et al. presented further two theories of entropic forces (Buskermolen et al., 2019) and gap avoidance (Buskermolen et al., 2020) to explain the contact guidance. In the “gap avoidance” theory, they considered the contact guidance as the result of “the energetic penalty of cell adhesions on non-adhesive gaps” (Buskermolen et al., 2020). Conceivably, contact guidance and cell bridges emerged simultaneously in the same culture system comprising of topographic and micropattern substrates. The molecular and biophysical mechanisms underlying these theories of cell bridges may complement the hypotheses about contact guidance; and hence, can be referred for a molecular and mechanistic understanding.

## The Crucial Factors Involved in the Maintenance of Cell Bridges

Actin edge-bundles are utilized in the cells’ maintenance and morphology (Zand and Albrecht-Buehler, 1989). Concave actin edge-bundles are constantly observed in suspended cell bridges. Various tissue- and cell-level variables are tightly coupled during the stabilization of cell bridges, such as geometry of ECM, inherent mechanical characteristics of cell monolayer, cross-talk between cell–cell and cell-ECM adhesions, and transmission and generation of force. For the stabilization of cell bridges on chemical micropatterns, Rossier et al. (2014) suggested that: (1) at the concave edges of single-cell bridges, the myosin-IIA triggers the assembly of actin filament at adhesion and (2) in the body of these bridges, myosin cross-links actin filaments and promote the healing of acto-myosin network when breaks occur. Furthermore, the actomyosin contractile force required in the bridged regions of cells spreading over non-adhesion areas involves the activation of the Rho-ROCK (Rho: a member of the Ras super family of low molecular weight GTPases; ROCK: Rho associated coiled coil forming protein kinase) pathway (Suffoletto et al., 2015). Conversely, myosin contractility is not necessary for the multicellular bridges, and the intermediate pool of E-cadherins is responsible for the stabilization of epithelial bridges on fibronectin micro-trips (Vedula et al., 2014b). Mitosis can also induce the destruction of epithelial bridges integrity in part (Vedula et al., 2014b). For multicellular bridges on topographic substrates with negative curvature, tissue contractility plays a crucial role in the detachment and formation of epithelial bridges, which involves ROCK, Rho GEFs (guanine nucleotide-exchange factors), the Ras (any of a family of genes that undergo mutation to oncogenes and especially to some commonly linked to human cancers)–Raf (a member of mitogen-activated protein kinase kinase kinase)–MEK (ERK kinase)–ERK (extracellular signal-regulated kinase) and Ras–PI3K (phosphatidylinositol-3- kinase) pathways (Broaders et al., 2015).

## The Influence of Detachment on Cells

An adhesive substrate must be approved for the anchorage-dependent cell to support its growth. Cells have to span over non-adhesive gaps due to the heterogeneity of ECM and microenvironment. Various cell bridges on chemical micropattern and topographic substrates have been observed and investigated. The impact of detachment and suspended status on the behavior and fate of cells is crucial for embryonic development, tissue healing, and scaffold preparation. A single cell or multicellular bridges are held by adhesive forces and are subjected to enormous tensions, and the force have been measured by researchers through various approaches. In 2010, using a PDMS pillar device, Rossier et al. (2014) measured the force generated by single-cell bridges and reported the largest traction forces of 5∼20 nN/pillar located in the vicinity of the concave edges. Hua et al. also revealed higher tension in bridging portions of cells using fluorescence resonance energy transfer (FRET) (Suffoletto et al., 2015). Vedula et al. (2014b) demonstrated the homogeneous landscapes of both velocity and vorticity in keratinocytes sheets with pluricellular bridges and heterogeneous landscapes in MDCK cells sheet with multicellular bridges by using particle image velocimetry. The results reflected the differences in traction force fields of both cell sheets and suggested that the keratinocytes exhibited elastic-like behavior, and MDCK indicated fluid-like behavior. Simultaneously, the stiffness of hMSCs bridges was roughly measured and compared by atomic force microscopy (AFM), and there was no difference among cell bridges across grooves with different width and normal spreading cells (Zhang et al., 2015b). In contrast, the lateral direction of epithelial bridges showed higher modulus in a diverging channel (Hu et al., 2017). Furthermore, cells exhibit decreased spreading and proliferation when they span over nanogratings with a height of 560 nm or partially attached on the bottom compared with cells cultured on 150 nm height pillars; however, the influence of height can be alleviated by the increasing nanogratings space (Song et al., 2016). Furthermore, the bridging behavior did not trigger the expression of desmin, osteocalcin, alkaline phosphatase (ALP), and alizarin in hMSCs (Zhang et al., 2015b). Notably, the cell bridges are tightly coupled with cell migration (Albert and Schwarz, 2016b) and wound repair (Hu et al., 2017). Although chemical micropattern and topographic cues on substrates can regulate cell fate (Anselme et al., 2010; Jing et al., 2016) through stressing effect on focal adhesion (Abagnale et al., 2015), cell shape, cytoskeletal tension, and RhoA signaling (McBeath et al., 2004), there is a paucity of studies on the mechanism and effect of cell bridges phenomenon on properties of cells, such as stiffness, proliferation, and differentiation.

## Conclusion and Outlook

Recent advances in the development of microtechnologies led to an increased understanding of cell bridges; however, similar to contact guidance, cell bridging as an optimum alignment of the cell should be investigated thoroughly. In our view, further aspects to consider includes the following: (1) a comprehensive investigation of cell bridges on topographic or chemical surface patterning should be performed to probe the effect of variable factors on cell bridges formations; (2) cell bridges and contact guidance usually co-exist, therefore, their interaction and synergies should be investigated carefully; (3) most of work on cell bridges are based on epithelial cells or keratinocytes, thus, cell bridges constitution in other cell types should be paid more attention, particularly stem cell on scaffolds, which is crucial for the regeneration medicine; (4) mechanistic studies of cell bridges at molecular-, subcellular-, cellular- and tissue-level should be performed using advanced techniques, such as traction force microscopy, particle image velocimtry (Vedula et al., 2014b), FRET (Suffoletto et al., 2015), complementary microscopic techniques (Fritzsche et al., 2017), second harmonic generation, and vibrational microscopy (Liu et al., 2017); (5) theoretical models should be adopted to describe and predict cell bridges, which will be helpful in understanding the role of cell bridges in cell–cell arrangement and organization, tissue regeneration, and shape problems in biological systems.

## Author Contributions

The author confirms being the sole contributor of this work and has approved it for publication.

## Conflict of Interest

The author declares that the research was conducted in the absence of any commercial or financial relationships that could be construed as a potential conflict of interest.
